# A Splice Site Mutation Associated with Congenital CD59 Deficiency

**DOI:** 10.3390/hematolrep14020025

**Published:** 2022-05-27

**Authors:** Jiani N. Chai, Abul Kalam Azad, Kevin Kuan, Xiaoling Guo, Yanhua Wang

**Affiliations:** Department of Pathology, Montefiore Medical Center, The University Hospital for Albert Einstein College of Medicine, 111 East 210th Street, New York, NY 10467, USA; jichai@montefiore.org (J.N.C.); aazad@montefiore.org (A.K.A.); kkuan@montefiore.org (K.K.); xguo@montefiore.org (X.G.)

**Keywords:** congenital CD59 deficiency, splice site mutation, atypical hemolytic uremic syndrome, next-generation sequencing (NGS), CD59

## Abstract

Congenital CD59 deficiency is a recently described rare autosomal recessive disease associated with *CD59* gene mutations that lead to deficient or dysfunctional CD59 protein on the cell surface. The disease is characterized by the early onset of chronic hemolysis, relapsing peripheral demyelinating neuropathy, and recurrent ischemic strokes. To date, there are 14 patients with 4 exon mutations reported globally. A young boy with early onset peripheral neuropathy and atypical hemolytic uremic syndrome is presented. Next-generation sequencing (NGS) identified a homozygous splice site variant in intron 1 of the *CD59* gene (c.67 + 1G > T). This variant alters a consensus donor splicing site. Quantitative reverse transcription PCR showed that *CD59* mRNA expression in the patient is significantly reduced to 0.017-fold compared to the controls. Flow cytometry showed the lack of CD59 protein on the surface of the patient’s red blood cells. This variant is the first splice site mutation reported to be associated with congenital CD59 deficiency.

## 1. Introduction

CD59 glycoprotein is an essential complement regulatory protein that protects cells against complement attack by inhibiting the membrane attack complex (MAC). MAC is a protein complex formed on the cell membrane surface following the activation of the complement system. It is composed of complement proteins C5b, C6, C7, C8, and C9 that sequentially bind to one another to form a pore in the plasma membrane and can cause cell lysis. CD59 protein binds to C8 and blocks the incorporation of C9 into MAC, thus preventing host cells from MAC-mediated cell injury [1]. CD59 is expressed widely on the membranes of human cells including erythrocytes, leukocytes, endothelial cells, Schwann cells, and neurons [2,3]. The deletion of the *CD59* gene has been shown to cause intravascular hemolysis, endothelial damage, and enhanced demyelination and axonal injury in experimental animal models [4,5,6]. 

CD59 is attached to the cellular membrane via a glycosylphosphatidylinositol (GPI) anchor. An acquired defect in the biosynthesis of GPI anchor can lead to the deficiency of CD59 protein on the cell surface and other GPI-anchored membrane proteins. This is seen in patients with paroxysmal nocturnal hemoglobinuria (PNH), which is due to a defect in phosphatidylinositol glycan A (PIGA), one of several enzymes needed to make GPI. In this condition, PIGA gene mutation occurs in hematopoietic stem cell and affects all mature blood cells derived from the abnormal stem cells. Patients with PNH experience a broad range of signs and symptoms, including anemia, dyspnea, abdominal pain, and thrombosis [7]. 

In recent years, a congenital CD59 deficiency associated with mutations in the *CD59* gene has been described. It is an extremely rare autosomal recessive disorder that has been reported in 14 patients worldwide with an onset age of 1 month to 13 year (Table 1) [8,9,10,11,12,13,14,15,16]. The affected patients suffer from early onset chronic hemolysis, relapsing peripheral demyelinating neuropathy mimicking Guillain–Barré syndrome (GBS), or chronic inflammatory demyelinating polyradiculoneuropathy (CIDP), and recurrent ischemic strokes. There are five different mutations reported to date: p.Cys89Tyr, p.Asp49Val, p.Tyr29Asp, p.Asp49fs, and p.Ala41fs (Table 1). These mutations all involve the coding sequence of the *CD59* gene and result in either the loss of cell surface CD59 expression or expression of the dysfunctional mutant protein on cell membrane [17]. 

## 2. Case Report

The patient initially presented at 2 years of age with difficulty walking. He was diagnosed with GBS and treated with intravenous immunoglobulin (IVIg). His symptoms improved after IVIg with residual cavus and equinovarus deformities of the bilateral foot. He ambulates with the assistance of shoe braces. At the age of 3 years, the patient presented with vomiting, diarrhea, and decreased urine output. Laboratory tests showed impaired renal function requiring hemodialysis. Workup is also concerning for hemolysis with a drop in hemoglobin requiring blood transfusion, fragmented red blood cells, increased level of lactic acid dehydrogenase (LDH), thrombocytopenia, abnormal coagulation panel, and elevated liver enzymes. 

His phenotype was further characterized by molecular and flow cytometry studies. NGS genetic testing was performed at an external commercial laboratory. Quantitative reverse transcription PCR (RT-qPCR) was performed in-house to detect *CD59* mRNA expression in the patient’s blood specimen as compared to controls (Primer and probe sets: *CD59* Thermo Fisher Hs00174141_m1; *ABL* Qiagen 670113, part number IP-PF-000068). Flow cytometry analysis using anti-CD55-FITC and anti-CD59-PE antibodies was performed in-house to detect CD55 and CD59 expression on the surface of the patient’s red blood cells (RBCs). 

**Figure 1:**
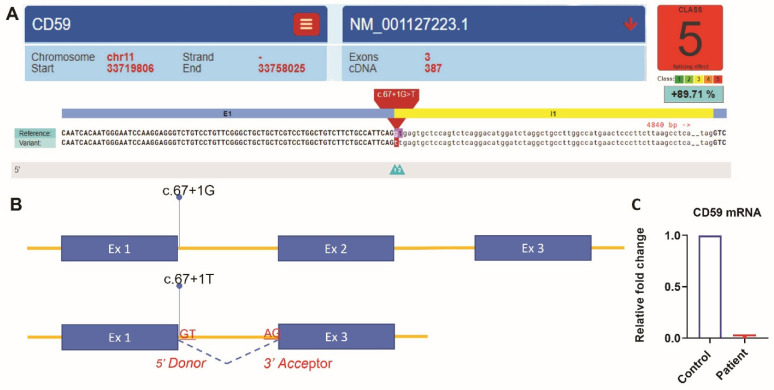
NGS analysis identified a homozygous splice site mutation c.67 + 1G > T in intron 1 of the *CD59* gene (NM_001127223.1). Spliceogenicity was assessed using the reference, alternate, and different maximum entropy scores thresholds. Variant within the native splice site was assessed for splice site loss using the native splice site scores [19]. (**A**) Variant effect predictor result was generated using (https://varseak.bio/index.php accessed on 16 September 2021). (**B**) Splicing effect of the variant c.67 + 1G > T. The mutant variant most likely abolishes the consensus splice site and produces the loss of function effect of the transcript by skipping exon 2. The splice site prediction class 5 with a positive score of 89.71% predicted the highest loss of function effect by https://varseak.bio/index.php (accessed on 16 September 2021). (**C**) Quantification of *CD59* mRNA expression by RT-qPCR. Relative fold change of *CD59* mRNA in the patient was normalized to the average expression in the controls using the delta–delta Ct method.

**Figure 2:**
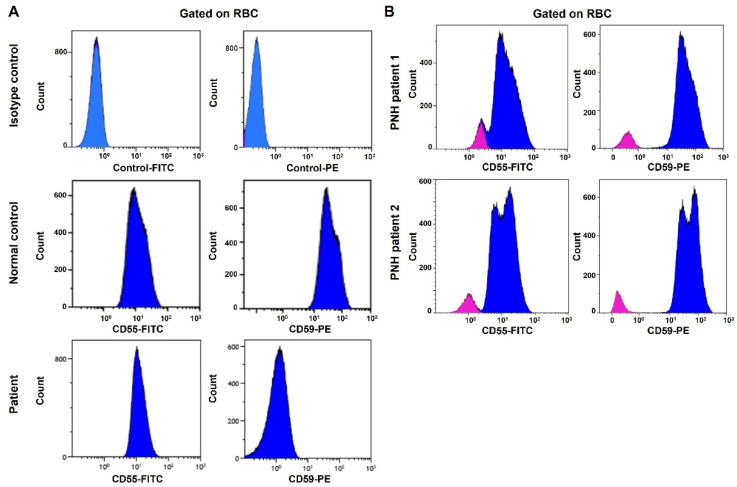
Flow cytometry analysis fails to detect CD59 protein expression on the surface of the patient’s RBCs. CD55 protein is expressed on the cell surface. (**A**) Top panel: RBCs stained with FITC and PE isotype control antibodies. Middle panel: RBCs from a normal control stained with anti-CD55-FITC and anti-CD59-PE antibodies. Bottom panel: RBCs from the patient stained with anti-CD55-FITC and anti-CD59-PE antibodies. (**B**) Flow cytometry analysis of CD55 and CD59 protein expression in PNH patients. RBCs from two PNH patients are stained with anti-CD55-FITC and anti-CD59-PE antibodies (Red: PNH clone; blue: normal RBCs).

Whole-exome sequencing was carried out using AmpliSeq Kit (Thermo Fisher Scientific, Waltham, MA, USA) with a coverage of 70–100X on an Ion S5™ XL next-generation sequencing (NGS) system. The NGS test identified a homozygous splice site mutation in intron 1 of the *CD59* gene (c.67 + 1G > T). This variant alters a consensus donor splicing site and is expected to be damaging by In Silico programs (Figure 1A,B). We then extracted RNA from the patient’s blood. Specimens from three subjects without any active hematologic diseases serve as controls. RT-qPCR showed that *CD59* mRNA expression in our patient is 0.017-fold decreased when compared to the average expression in the controls (Figure 1C). Flow cytometry analysis showed the lack of CD59 protein expression on erythrocytes, but the other GPI-linked antigen CD55 was present (Figure 2A). This is different from PNH, which has absent GPI-linked proteins, including both CD55 and CD59, on the PNH clone (Figure 2B). The characteristic clinical presentation of the patient is very similar to patients with pathogenic CD59 mutations. According to the American College of Medical Genetics (ACMG) guidelines [18], this novel canonical splice site variant in a gene where the loss of function is a known mechanism of disease can be classified as “pathogenic”. The patient’s family is of Middle Eastern origin and was not tested for the identified mutation. However, no other direct family members show any signs or symptoms of CD59 deficiency.

The patient was diagnosed with atypical hemolytic uremic syndrome (aHUS; OMIM #107271) associated with congenital CD59 deficiency. Anti-C5 monoclonal antibody eculizumab therapy was initiated and his hemoglobin, platelet count, LDH level, and liver function have since normalized. However, his renal function remained abnormal. He continued to be on hemodialysis twice a week before receiving a deceased-donor kidney transplant at the age of 5 years. At the time of this report, the patient is asymptomatic. He is on eculizumab infusion every two weeks, as well as tacrolimus, prednisone, and mycophenolate mofetil therapy. 

## 3. Discussion and Conclusions

The *CD59* gene consists of 7 exons and 6 introns spanning 33470 base pairs of DNA on chromosome 11 in humans (NG_008057.1). All five *CD59* mutations previously reported are located in the exons, including three missense mutations p.Cys89Tyr, p.Asp49Val, and p.Tyr29Asp, and two frameshift mutations p.Asp49fs and p.Ala41fs. It was shown recently that the missense mutants p.Cys89Tyr and p.Asp49Val generate nonfunctional cell surface proteins, whereas proteins transcribed from the frameshift mutants p.Asp49fs and p.Ala41fs do not reach the cell surface; they are secreted or degraded via the ubiquitin–proteasome pathway [17]. Congenital CD59 deficiency is an autosomal recessive disease, and each parent of an affected patient is usually a heterozygous carrier. However, several publications have reported mutations that appeared to be homozygous yet had another underlying cause, such as uniparental isodisomy (UPD) and area of homozygosity (AOH) or loss of heterozygosity (LOH) [20].

This report is the first to have a *CD59* mutation in the intron region. NGS analysis identified a homozygous splice site mutation in intron 1 of the *CD59* gene (c.67 + 1G > T). This variant altered a consensus 5′ donor splicing site (GT) and is expected to be damaging by In Silico programs. A mutation in the splice site may lead to the retention of large segments of introns or skipping of exon/exon fragments during the pre-mRNA splicing. These changes could result in the production of a nonfunctional CD59 protein or premature mRNA degradation [21]. RT-qPCR showed that *CD59* mRNA expression in our patient is significantly reduced compared to the controls. Furthermore, we did not detect CD59 protein expression on the erythrocytes’ surface by flow cytometry. Therefore, our data suggested this splicing site likely resulted in premature mRNA degradation and lack of mutant product expression on the cell surface. These findings are consistent with the patient’s clinical presentation of absent CD59 protein expression. 

To date, most of the reported congenital CD59 deficiency cases, including our patient, have signs and symptoms of hemolytic anemia and peripheral neuropathy (14 out of the 15 cases). Cerebral infarctions are seen in 7 out of the 15 cases. In addition to hemolytic anemia and peripheral neuropathy, the patient reported in this paper developed severe renal dysfunction. Hemolytic-uremia-like syndrome followed by spontaneous normalization of renal function has been reported in previous cases. Our case is the first reported to have permanent renal dysfunction requiring kidney transplantation. 

Eculizumab is a humanized monoclonal antibody with a high binding affinity for the human complement protein C5. It inhibits the formation of the MAC by preventing the cleavage of C5 to C5a and C5b. Eculizumab has been used successfully in patients with PNH, aHUS, and neuromyelitis optica [22,23,24]. In recent 10 years, it is also applied to treat congenital CD59 deficiency and shows positive response in alleviating the symptoms of hemolysis and neurologic disorder in all the reported cases, including our case [11]. Follow-up of these patients will help to assess its efficacy and safety in long-term use. In addition to eculizumab, emerging therapeutic technologies, such as small molecule intervention or gene editing targeting the mutant CD59 gene, are promising areas worth investigating [25].

In summary, we report the first case with a splice site mutation in the intron 1 of the *CD59* gene that is associated with congenital CD59 deficiency. This novel canonical splice site variant in a gene where the loss of function is a known disease mechanism is considered to be pathogenic. This report highlights the importance of combining molecular testing and flow cytometry in diagnosing suspected patients with symptoms of early onset hemolytic anemia, periphery neuropathy, and ischemic stroke. 

## Figures and Tables

**Table 1:** Literature review of the published variants in the *CD59* gene. Variants are listed in the order of nucleotide position.

Mutation ^‡^	Age of Onset	Gender	Clinical Manifestations				Reported Year
			Hemolytic Anemia	Peripheral Neuropathy	Cerebral Infarction	Hemolytic-Uremia-like Syndrome	
c.85T > G; p.Tyr29Asp (point mutation, missense)	15 mo	Female		Yes			2020 [16]
1-BP DEL, CODON 16; p.Ala41fs (deletion, frameshift, stop codon)	13 yr	Male	Yes		Yes		1990 [14,15]
c.67 + 1G > T (splice site mutation, exon skipping)	2 yr	Male	Yes	Yes		Yes	Current
c.146del; p.Asp49fs (deletion, frameshift, stop codon)	7 mo	NA	Yes	Yes	Yes	Yes	2014 [11]
	1 mo	Female	Yes	Yes	Yes		2017 [12]
c.146A > T; p.Asp49Val (point mutation, missense)	11 mo	Female	Yes	Yes	Yes		2015 [13]
	6 mo	Female	Yes	Yes	Yes		
	6.5 mo	Male	Yes	Yes			
c.266G > A; p.Cys89Tyr (point mutation, missense)	3.5 mo	Male	Yes	Yes		Yes	2013 [8]
	7 mo	Female	Yes	Yes			
	3 mo	Male	Yes	Yes			
	3 mo	Male	Yes	Yes			
	4 mo	Female	Yes	Yes			
	5 mo	Male	Yes	Yes	Yes	Yes	2015 [9]
	3 mo	Female	Yes	Yes	Yes		

^‡^ All the published variants are homozygous mutations.

## Data Availability

The data presented is contained within the current article.
